# Anti-hyperglycemic effects of three medicinal plants in diabetic pregnancy: modulation of T cell proliferation

**DOI:** 10.1186/1472-6882-13-77

**Published:** 2013-04-08

**Authors:** Akadiri Yessoufou, Joachim Gbenou, Oussama Grissa, Aziz Hichami, Anne-Marie Simonin, Zouhair Tabka, Mansourou Moudachirou, Kabirou Moutairou, Naim A Khan

**Affiliations:** 1 Laboratory of Cell Biology and Physiology, Department of Biochemistry and Cellular Biology, Faculty of Sciences and Techniques (FAST) and Institute of Applied Biomedical Sciences (ISBA), University of Abomey-Calavi, Cotonou, 01 BP 918, Benin; 2 Laboratory of Pharmacognosy and Essential Oils (ISBA/FAST), University of Abomey-Calavi, Cotonou, 01 BP 918, Benin; 3 Department of Physiology and Functional Exploration, University Hospital Farhat Hached, Sousse, 4000, Tunisia; 4 INSERM U866, University of Bourgogne, 6 BD Gabriel, Dijon, 21000, France; 5 University of Bourgogne, Physiology of Nutrition and Toxicology, INSERM UMR866, AgroSup/UB, Faculty of Life Sciences, 6 Boulevard Gabriel, Dijon, 21000, France

**Keywords:** Medicinal plants, Diabetic pregnancy, Antioxidants, T-cell proliferation, Fatty acids

## Abstract

**Background:**

Populations in Africa mostly rely on herbal concoctions for their primarily health care, but so far scientific studies supporting the use of plants in traditional medicine remain poor. The present study was undertaken to evaluate the anti-hyperglycemic effects of *Picralima nitida* (seeds), *Nauclea latifolia* (root and stem) and *Oxytenanthera abyssinica* (leaves) commonly used, in diabetic pregnancy.

**Methods:**

Pregnant wistar rats, rendered diabetic by multiple low injections of streptozotocin, were treated with selected plant extracts based on their antioxidant activities. Vitamin C concentrations, fatty acid compositions and phytochemical analysis of plants extracts were determined. Effect of selected plant extracts on human T cell proliferation was also analysed.

**Results:**

All analysed plant extracts exhibited substantial antioxidant activities probably related to their content in polyphenols. *Picralima nitida* exhibited the highest antioxidant capacity. Ethanolic and butanolic extracts of *Picralima nitida*, butanolic extract of *Nauclea latifolia* and ethanolic extract of *Oxytenanthera abyssinica* significantly decreased hyperglycemia in the diabetic pregnant rats. Butanolic extract of *Picralima*, also appeared to be the most potent immunosuppressor although all of the analysed extracts exerted an immunosuppressive effect on T cell proliferation probably due to their linolenic acid (C18:3n-3) and/or alkaloids content. Nevertheless, all analysed plants seemed to be good source of saturated and monounsaturated fatty acids.

**Conclusion:**

By having antioxidant, anti-hyperglycemic and immunosuppressive activities, these plants could be good candidates in the treatment of diabetes and diabetic pregnancy.

## Background

The prevalence of diabetes mellitus (DM) is increasing across the world and in the year 2011 the World Health Organization [1] estimated that over 346 million of people live with DM worldwide. Nearly 80% of deaths due to DM occur in low and middle income countries. Because of the high cost of conventional treatments with synthetic drugs, traditional treatment with plants becomes an alternative option for financially poor populations who may have problems of accessibility to modern drugs. For these reasons, the development of new therapies from plants that are able to control diabetes mellitus is of great interest. Several plants have been used by traditional and ancestral medicine men in African countries for the treatment of several pathologies including digestive disorders, weakness, liver complaints, obesity, urinary troubles, diabetes, skin infections, fever, diarrhoea and insomnia [2-6]. However, the use of some of the plants still suffers from the lack of scientific evidence which may support their inclusion in the treatment of certain diseases like diabetes. We have recently investigated the role of crude extracts of different parts of *Zizyphus lotus* L (Desf.), a plant commonly used in the Mediterranean traditional medicine for its anti-diabetic properties, sedative, analgesic, anti-inflammatory and hypoglycaemic activities [7,8], on human T cell proliferation [9]. In the present study, we report the results of three plants: *Nauclea latifolia, Oxytenanthera abyssinica* and *Picralima nitida* commonly and traditionally used in Benin, Africa by diabetic patients.

*Nauclea latifolia* belongs to Rubiaceae family and is a small tree found in tropical areas in Africa. It is used in traditional medicine to treat malaria, epilepsy, anxiety, pain, fever [5,6]. Moreover, root stem of this plant is traditionally and empirically used by diabetic patients in Benin to manage their glycemia.

*Oxytenanthera abyssinica* (Gramineae), commonly known as bamboo, is a plant found in tropical areas in Africa. This plant has a short rhizome and a rapid growth (over 20 mm per day) and can reach 10 m height with 5 cm of diameter [10]. Traditionally, decoction of plant leaves is used in the treatment of diabetes in Senegal and Togo.

*Picralima nitida* (Stapf.) (Apocynaceae) is widely found in Western Africa, from Côte d’Ivoire to Uganda and further to south of Democratic Republic of Congo and Cabinda region in Angola. Several studies have reported that different parts of this plant (seeds, root, stem) possess many properties and is used as an anti-malaria, antimicrobial, anti-inflammatory and analgesic [11,12]. Several recent studies have also demonstrated anti-ulcer activity of methanolic extract of the plant seeds [13]. Seeds of this plant have, from time in memorial, been consumed by diabetic patients in some West African countries to control their glycemia. While few studies have reported that extract from the seeds of this plant did not possess antidiabetic and hypoglycemic activities [14] in contrast, other studies have proved the antidiabetic activity of the seed extract in alloxan-induced diabetic animals [15,16].

Most of these studies cited above reported the activities of plants in several pathologies. However, none of them has investigated their efficacy in diabetes associated with pregnancy. Convincing evidence has shown that diabetic pregnancy, defined as either maternal pre-existing diabetes (type 1 and type 2 diabetes) or gestational diabetes mellitus (which occurs only during pregnancy), is associated with macrosomia. Indeed, we have previously demonstrated, in many clinical and experimental studies, that diabetes during pregnancy is an important risk factor for fetal overnutrition and macrosomia, and for the development of obesity and diabetes in adult offspring [17-22]. In fact, experimental diabetes during pregnancy, induced by a high single dose of streptozotocin, occurs by direct toxic effects on pancreatic β-islet cells [23]. The fetal growth is retarded, leading to fetal microsomia (low birth weight) [23]. Postnatal development is also retarded, and the offspring remain small at adulthood; and they develop insulin resistance [23]. Streptozotocin, administered at low doses during 5 consecutive days, induces mild type 1 diabetes, following a T-lymphocyte-dependent process, an autoimmune destruction of pancreatic β cells [24]. This model of diabetes during pregnancy leads to macrosomia in offspring. Macrosomic (large-sized) offspring of diabetic dams maintained an accelerated weight gain until adulthood [17-22].

Moreover, all forms of diabetic pregnancy have been linked to a pathological role of immune system and inflammation [24-26] which implicates T-lymphocytes, the principal mediators of immune-mediated pathologies. Hence, an intervention on T cell activation would be a valuable tool to disrupt disease progression. Since the plants reported in this study have been shown to modulate different disorders, we then aim to investigate their efficacy in diabetic pregnancy in rats and their action through the modulation of T cell proliferation. We also examine antioxidant capacity of the plant extracts, since antioxidants have been reported to modulate immune system [27].

## Methods

### Plant materials’ collection and extracts’ preparation

Plants were collected from the south-eastern part of Benin (Abomey-Calavi, Adjarra, Agonvy in Departments of Atlantic and Oueme) between half July to half August 2007 during the short dry season when the mean temperature equals to 28 ± 2°C (Agence de Sécurité de la Navigation Aérienne, Station de Dangbo). This period is preceded by the great rain season (half March to half July). The area belongs to hydromorphic lateritic soil on clay sediments (reference: Carte pédologique de reconnaissance à 1/200,000 Feuille de Porto-Novo 1975, Benin) and these plants adapt to this kind of soil. Plants were identified by the Principal Botanist of National Herbarium of Benin of University of Abomey-Calavi, where the voucher specimens were deposited under following numbers: *Picralima nitida* (Stapf.) Apocynaceae T & H Durand: AP-1904-HNB; *Nauclea latifolia* Rubiaceae: AP-2081-HNB and *Oxytenanthera abyssinica* Gramineae (A. Rich) Munro: AP-2076-HNB.

Parts of plants including the seeds of *Picralima nitida*, root and stem of *Nauclea latifolia* and leaves of *Oxytenanthera abyssinica* were collected and used to prepare different extracts as described elsewhere [28]. The plant materials were dried at laboratory air-conditioned temperature and stored in a dry place prior to use. Materials were reduced into powder and subjected to five consecutive liquid-liquid extractions to obtain five different fractions. Briefly, 100 g of powder were suspended in 500 ml of ethanol 96% and mixed for 72 h. The collected filtrate was evaporated under vacuum at 50°C to drive away alcohol. The ethanolic extract (a) was obtained and subjected to liquid-liquid extraction. A part of ethanol-extract was added to distilled water and diethyl-ether (volume/volume). This solution was macerated and showed two phases: aqueous phase and ether phase. The ether phase was evaporated under vacuum at 50°C to obtain Ether-extract (b). The collected aqueous phase was added to ethyl-acetate (volume/volume). The solution was mixed and also showed two phases: aqueous and ethyl-acetate phases. Ethyl-acetate-extract (c) was then obtained by evaporating under vacuum at 50°C and the collected aqueous phase was also added with n-butanol. The solution was mixed and also showed two phases: aqueous phase and butanolic phase. The butanolic phase was then evaporated under vacuum at 50°C in order to obtain butanolic extract (d). The residual aqueous phase was then lyophilized and constituted residual aqueous extract (e). All the obtained extracts were then used for study.

### Diabetes induction and animal treatment by plant extracts

Female Wistar rats from 2 to 3 month old, weighing from 200 to 250 g, were obtained from IFA-CREDO (Abresle, France). After 1 week of acclimation, animals were divided into experimental groups. After mating, the first day of gestation was estimated by the presence of spermatozoids in vaginal smears. Pregnant rats were housed individually in wood chip-bedded plastic cages at constant temperature (25°C) and humidity (60 ± 5%) with a 12-hour light/dark cycle.

Each experimental group consisted of ten pregnant rats. For diabetes induction, pregnant rats were rendered diabetic by intraperitoneal administration of five low doses of streptozotocin (20 mg/kg body weight, in 0.1 M citrate buffer, pH 4.5), starting on the fifth day of gestation (day5 to day9) [19-22,24,25]. Control pregnant animals were injected with the vehicle alone and considered as a control group.

Then, plant extracts diluted in sterile physiological saline solution (NaCl 0.9%) were sterilized and filtrated [filter 0.20 μm (Nalge Nunc International Corp., USA)] and injected intraperitoneally for four consecutive days to diabetic or control animals (25 mg/kg body weight), from day12 to day15 of gestation. As positive controls, one group of diabetic animals was injected with four consecutive doses of insulin (0.75 U/kg body weight; Actrapid Novo, Copenhagen, Denmark).

For the determination of glycemia during gestation maternal blood was collected after an overnight fasting on day12 and day16 of gestation by cutting off the tip of the tail and squeezing it gently. Fasting glycemia was measured using One Touch ULTRA ^®^Glucometer (LifeScan, Johnson and Johnson, USA). At delivery on day21 and after overnight fasting, animals were anaesthetized with pentobarbital (60 mg/kg body weight), as described previously [19-22]. The abdominal cavity was opened, and whole blood was drawn from the abdominal aorta. Plasma was obtained by low-speed centrifugation (1000 g, 20 min) and immediately used for glucose and vitamin C determinations. The general guidelines for the care and use of laboratory animals, recommended by the Council of European Economic Communities, were followed. The experimental protocol was approved by the Regional Ethical Committee (Comité d’Ethique de l’Expérimentation Animale of University of Bourgogne, Dijon, France; Researcher Authorization number n °: 21 CAE 069)”.

### Determination of vitamin C concentration in plant extracts and plasma

Total ascorbate (vitamin C) levels were determined in lyophilised plant extracts (100 mg/ml in phosphate buffered saline, pH 7.4) or in rat plasma on day21 (at delivery) using the method of Roe and Kuether [29] as described below. After protein precipitation in extract supernatant or in plasma with 10% trichloroacetic acid and centrifugation, the supernatant (500 μl) was mixed with 100 μl of DTC reagent (9 N sulfuric acid containing 2,4-dinitrophenylhydrazine 3%, thiourea 0.4% and copper sulfate 0.05%) and incubated at 37°C for 3 h. After the addition of 750 μl of 65% (v/v) sulfuric acid, the absorbency was recorded at 520 nm.

### Antioxidant capacity of plant extracts

Plant extract resistance to free radical aggression was used to determine the capacity of red blood cells (RBCs) to withstand free radical-induced haemolysis and was measured according to the method of Blache and Prost [30] who have demonstrated that if at least one component of the antiradical detoxification system (antioxidants, enzymes) is impaired a shift of the haemolysis curve is observed toward shorter times. Briefly, washed RBCs were diluted (1:40, v/v) with anti-radical resistance [(Kit Radicaux Libres (KRL^®^; Kirial International SA, Couternon, France)] buffer (300 mOsmol/kg) and 50 μL of RBCs suspension was assayed in a 96-well microplate coated with a free radical generator (GRL, Kirial International SA). The kinetic of RBCs resistance to haemolysis in the presence of plant extract solutions (1 mg/ml), was determined at 37°C by continuous monitoring of changes in absorbance at 620 nm. The time to reach 50% of total haemolysis was retained for group comparisons.

### Phytochemical analysis of the extracts

Chemical compounds of the total brut extracts of plants (Table 1) were investigated using the methods of Houghton and Amala [31] based on colorimetric reactions and differential precipitations.

**Table 1:** Phytochemical composition of plants

**Chemical compounds class**	**Test**		**Plant name (part used)**		
			***Nauclea latifolia *(root and stem)**	***Picralima nitida*(seeds)**	***Oxytenanthera abyssinica *(leaves)**
**Alkaloids**	General test: Dragendorff reagent		+++	-	+++
	Extraction: Mayer reagent		+++	++	+++
**Gallic tannins**	Saturation of Na acetate + a few drips of FeCl3, 1%		+	+++	++
**Cathechic tannins**	Stiasny reagent		-	-	+++
**Flavonoids**	Shinoda reagent (cyanidine reaction)		-	++	++
**Anthocyanes**	Adding some drips of HCl 5% to 1 mL of decocted + alcalinisation (with drips of ammoniac 50%)		-	+	+
**Leucoanthocyanes**	Shinoda reagent (chlorhydric alcohol)		-	-	+
**Quinonic derivates**	Born-Trager reaction	Concentrated HCl	++	+	-
		Diluted HCl	+++	++	-
**Saponosides**	Foam index (FI) of diluted aqueous decoction (positive if FI ≥ 100, meaning foam height ≥ 1 cm)		+++	-	+
			(FI > 1 cm)	(FI < 1 cm)	(FI = 1 cm)
**Triterpenoids**	Liebermann-Buchard reaction (acetic anhydride-sulfuric acide 50 :1)		-	-	-
**Steroids**	Kedde reaction (dinitrobenzoic acid 2% in ethanol + NaOH (1 N) 1:1)		-	-	+++
**Cardenolids**	RAYMOND reaction (Dinitrobenzene 1% in ethanol + NaOH 20%)		-	-	-
**Cyanogenic derivates**	Grignard reaction (soaked paper with picric acid 5%		-	-	-
**Mucilages**	Viscosity study (in absolute ethanol)		+++	++	-

Chemical compounds of total brut extract of selected parts of plants. The phytochemical analysis was performed as described in Methods section. (+++) too high, (++) high (+) low: indicates the presence of the compounds in the plants; (−) indicates the absence of compound in plants.

### Effects of plant extracts on T cell blastogenesis

Human (Jurkat) T-cells were routinely cultured in RPMI-1640 medium supplemented with 10% foetal calf serum, 2 mM L-glutamine, 100 U/ml of penicillin, and 100 μg/ml of streptomycin and 25 mM HEPES at 37°C in a humidified chamber containing 95% air and 5% CO_2_. Cell viability was assessed by trypan blue exclusion test. Cell numbers were determined by hemocytometer.

To investigate the effects of plant extracts on T cell proliferation, Jurkat T cells (0.1x10^6^ cells/160 μl) were suspended in RPMI-1640 without serum and seeded in 96-well plate (Nunc, Roskilde, Denmark), then cells were incubated for 46 h with increasing concentration of plant extract solutions (5 μg.ml^-1^, 10 μg.ml^-1^ and 20 μg.ml^-1^), then stimulated with anti-CD3 antibody (1 μg/well). Cells were distributed in six replicates as follows: 160 μl of cell suspension, 20 μl of sterile and filtrated plant extract and 20 μl of anti-CD3 antibodies, as previously described [9]. Plates were incubated for 46 h at 37°C in a 5% CO_2_/air atmosphere. For the last 6 hours, 0.8 μCi/well of (^3^H)-thymidine in 20 μL of RPMI-1640 medium was added.

At the end of the incubation period, cells were collected using a cell harvester (Dynatech, Burlington, MA, USA), trapping their DNA onto glass filter-mats. When the filter circles were dried, they were placed in plastic mini-vials (Packard, Downers Grove, IL, USA) and added with 4 ml Optifluor-O (Packard). The radioactivity was recorded in a scintillation counter (Beckman, Fullerton, CA, USA).

### Determination of fatty acid composition in plants extracts

The details of the fatty acid composition of plant extracts are presented in Table 2. Total lipids were extracted from solutions of lyophilized plant extracts (1 mg/ml in physiological saline solution) according to the method of Bligh and Dyer [32], then transmethylated by BF3/methanol after saponification, and fatty acids were analysed by gas liquid chromatography [33], using C17:0 as internal standard, with a Becker gas chromatograph (Becker instruments, downers grove IL) equipped with a 50 m capillary glass column packed with carbowax 20 m (Spiral-RD, Couternon, France). Analysis of fatty acid peaks was achieved with reference to the internal standard by using DELSI ENICA 21 integrator (Delsi Nermag, Rungis, France).

**Table 2:** Per cent of total fatty acid composition of plant extracts

	**(A) Ethanolic extract**			**(B) Ether (Et2O) extract**			**(C) Ethyl acetate extract**			**(D) Butanolic extract**			**(E) Residual Aqueous extract**		
**Fatty acids (%)**	***P. nitida***	***N. latifolia***	***O. abyssinica***	***P. nitida***	***N. latifolia***	***O. abyssinica***	***P. nitida***	***N. latifolia***	***O. abyssinica***	***P. nitida***	***N. latifolia***	***O. abyssinica***	***P. nitida***	***N. latifolia***	***O. abyssinica***
C14:0	0.0	1.4 ± 0.1	3.6 ± 0.1*	1.2 ± 0.09	8.0 ± 0.5*	8.8 ± 0.5*	0.0	0.0	3.4 ± 0.7*	ND	ND	ND	ND	ND	ND
C16:0	23.8 ± 1.2	44.2 ± 2.6*	51.4 ± 3.1*	2.0 ± 0.1	36.3 ± 1.6*	38.9 ± 4.0*	22.9 ± 1.2	4.5 ± 0.6*	54.9 ± 4.1*	27.3 ± 1.2	40.5 ± 2.9*	42.1 ± 3.9*	29.9 ± 2.3	0.0*	70.5 ± 4.9*
C18:0	7.4 ± 0.6	15.4 ± 1.5*	12.5 ± 0.9*	22.2 ± 1.9	38.6 ± 2.8*	11.2 ± 0.8*	4.4 ± 0.1	10.6 ± 1.2*	6.8 ± 0.6	9.5 + 1.2	22.6 ±1.6*	22.1 ± 1.7*	10.2 ± 1.2	21.3 ± 2.1*	13.4 ± 0.9
**∑ SFA**	**31.2**	**61.0**	**67.5**	**25.4**	**82.9**	**58.9**	**27.3**	**15.1**	**65.1**	**36.8**	**63.1**	**64.2**	**40.1**	**21.3**	**83.9**
C16:1	3.7 ± 0.9	19.9 ± 1.5*	16.7 ± 0.8*	13.0 ± 1.1*	0.0*	3.2 ± 0.1*	2.8 ± 0.08	51.0 ± 3.5*	12.0 ± 0.4*	9.3 ± 0.5	29.6 ± 2.5*	32.4 ± 0.9*	27.0 ± 3.3	60.6 ± 5.7*	16.1 ± 1.1*
C18:1	61.6 ± 1.9	13.0 ± 1.0*	8.6 ± 0.4*	17.4 ± 1.1*	9.5 ± 0.7*	5.7 ± 0.9*	66.9 ± 1.6	22.4 ± 3.2*	10.2 ± 0.9*	52.0 + 3.8	5.5 ± 0.1*	1.9 ± 0.09*	32.9 ± 3.1	18.1 ± 1.0*	0.0
**∑ MUFA**	**65.3**	**32.9**	**25.3**	**30.4**	**9.5**	**8.9**	**69.7**	**73.4**	**22.2**	**61.3**	**35.1**	**34.3**	**59.9**	**78.7**	**16.1**
C18:2n-6 (LA)	ND	ND	ND	16.5 ± 1.0	7.6 ± 0.9*	15.9 ± 1.0	ND	ND	ND	ND	ND	ND	ND	ND	ND
C20:4n-6 (AA)	ND	ND	ND	ND	ND	ND	ND	ND	ND	ND	ND	ND	ND	ND	ND
**∑ n-6 PUFA**	ND	ND	ND	**16.5**	**7.6**	**15.9**	ND	ND	ND	ND	ND	ND	ND	ND	ND
C18:3n-3 (LNA)	3.6 ± 0.2	6.1 ± 0.9*	7.1 ± 0.9*	27.6 ± 2.5	ND	16.3 ± 1.1*	3.0 ± 0.2	11.5 ± 1.6*	12.8 ± 0.5*	1.9 ± 0.1	1.8 ± 0.2	1.5 ± 0.05	ND	ND	ND
C20:5n-3 (EPA)	ND	ND	ND	ND	ND	ND	ND	ND	ND	ND	ND	ND	ND	ND	ND
C22:6n-3 (DHA)	ND	ND	ND	ND	ND	ND	ND	ND	ND	ND	ND	ND	ND	ND	ND
**∑ n-3 PUFA**	**3.6**	**6.1**	**7.1**	**27.6**	ND	**16.3**	**3.0**	**11.5**	**12.8**	**1.9**	**1.8**	**1.5**	ND	ND	ND
**Total FA**	**100.0**	**100.0**	**100.0**	**100.0**	**100.0**	**100.0**	**100.0**	**100.0**	**100.0**	**100.0**	**100.0**	**100.0**	**100.0**	**100.0**	**100.0**

Fatty acid compositions were determined as described in Methods section. Values are mean ± SEM. Each value represents the mean of three determinations. * p < 0.05 indicated significant differences as compared to *Picralima nitida* extract. ND = not detectable. MUFA: monounsaturated fatty acids; SFA: saturated fatty acids; PUFA: polyunsaturated fatty acids.

### Statistical analysis

Values are mean ± SEM. Statistical analysis of data was performed. Data were evaluated by analysis of variance. Duncan's Multiple-Range test was employed for the comparison. Differences were considered significant when *p* < 0.05.

## Results

### Plant effects on glycemia and vitamin C levels during diabetic pregnancy in rats

**Figure 1:**
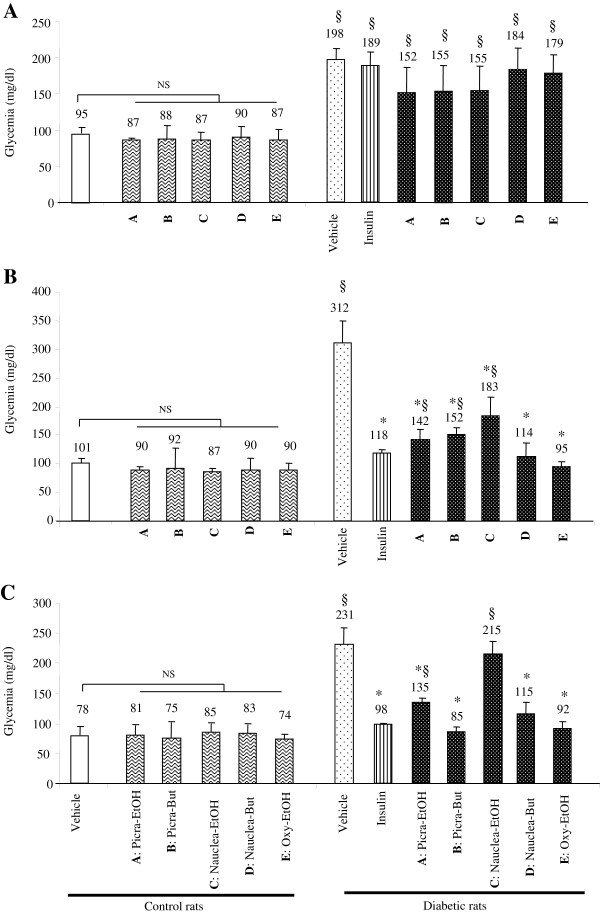
**Glycemia in diabetic pregnant rats treated with plant extracts on day12 (A), day16 (B) and day21 (C) of gestation.** Pregnant rats were rendered diabetic by administration of five low doses of streptozotocin, starting on day5 of gestation. Lyophilised plant extracts diluted in sterile physiological saline solution (NaCl 0.9%) were sterilized and filtrated [filter 0.20 μm (Nalge Nunc International Corp., USA)] and injected intraperitoneally to diabetic or control animals from day12 to day15 of gestation (four days). Values are mean ± SEM. (§) p < 0.01 = significant difference between diabetic rats compared with treated/untreated control animals. (*) p < 0.01 = significant difference between plant/insulin-treated diabetic rats compared with untreated diabetic animals. NS = non-significant difference. A: Picra-EtOH: ethanolic fraction of *Picralima nitida*; B: Picra-But: butaniolic fraction of *Picralima nitida*; C: Nauclea-EtOH: ethanolic fraction of *Nauclea latifolia*; D: Nauclea-But: butanolic fraction of *Nauclea latifolia*; E: Oxy-EtOH: butanolic fraction of *Oxytenanthera abyssinica*.

In the control animals treated with plant extracts, we observed that glycemia was at normal level during pregnancy (Figures 1A, 1B, 1C). However, in diabetic animals, *Picralima* and *Nauclea* butanolic extracts and *Oxytenanthera* ethanolic extract induced a significant decrease of glycemia in diabetic pregnant rats from day16 until delivery on day21 (Figure 1B, C). Nonetheless, *Picralima* ethanolic extract also significantly decreased the glycemia though its value remained moderately high on day21 in the diabetic animals (Figure 1C). The fifth extract, *Nauclea* ethanolic did not decrease glycemia in diabetic rats throughout pregnancy.

**Figure 2:**
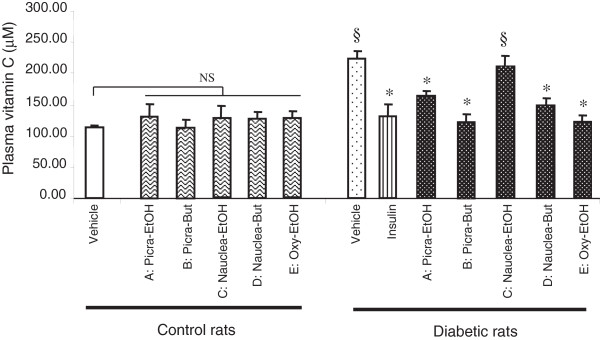
**Plasma vitamin C concentration in control and diabetic rats.** Vitamin C concentration was determined in the plasma of the control and diabetic animals at delivery (day21). Values are mean ± SEM. (§) p < 0.01 = significant difference between diabetic rats compared with treated/untreated control animals. (*) p < 0.01 = significant difference between plant/insulin-treated diabetic rats compared with untreated diabetic animals. NS = non-significant difference. A: Picra-EtOH: ethanolic fraction of *Picralima nitida*; B: Picra-But: butaniolic fraction of *Picralima nitida*; C: Nauclea-EtOH: ethanolic fraction of *Nauclea latifolia*; D: Nauclea-But: butanolic fraction of *Nauclea latifolia*; E: Oxy-EtOH: butanolic fraction of *Oxytenanthera abyssinica*.

As far as plasma vitamin C concentration is concerned, there was no significant difference in vitamin C concentrations among plant-treated control rats and untreated control animals (Figure 2). However, in the diabetic groups, highest level of vitamin C was observed in pregnant diabetic animals treated with vehicle or *Nauclea*-EtOH. Plasma vitamin C levels were significantly lower in diabetic animals treated with insulin, Picra-but, Nauclea-but, Oxy-EtOH, and Picra-EtOH extracts than in untreated diabetic rats, but did not differ when compared with control animals.

### Antioxidant capacity of plant extracts

**Figure 3:**
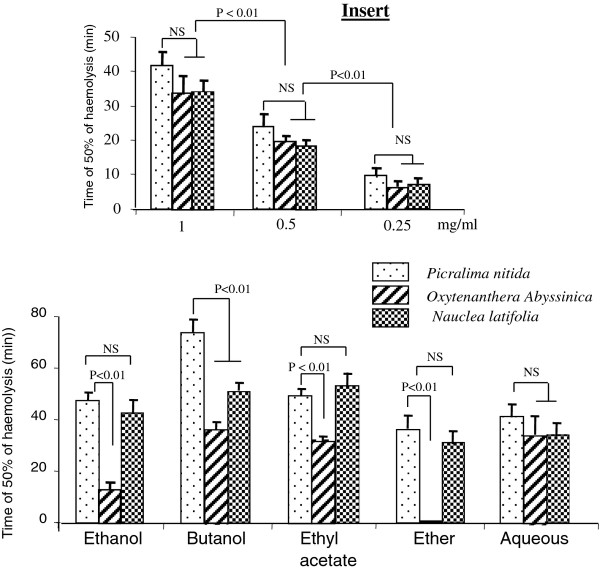
**Antioxidant capacity of plant extracts.** Antioxidant capacity of plant extracts was determined in a solution of 1 mg/ml as described in the Materials and Methods section. Results are mean ± SEM. Each value represents the mean of three determinations.

*Picralima* butanolic extract exhibited the highest antioxidant capacity; however in other extracts, this capacity was similar to that of *Nauclea* (Figure 3). *Oxytenanthera* showed appreciable and similar antioxidant activity in all extracts, except in ethanolic and ether extracts where it was respectively low and null. The order of the antioxidant capacity is:

*Picralima*: …………. butanolic > ethanolic = ethyl acetate = aqueous > ether

*Nauclea*: …………… butanolic = ethanolic = ethyl acetate > aqueous = ether

*Oxytenanthera*: ……. butanolic = ethyl acetate = aqueous > ethanolic > ether

The insert of Figure 3 represents the antioxidant activity of aqueous extracts of the plants. It shows that the antioxidant capacity of the plants was in a dose-dependent manner. Based on this antioxidant capacity, we have selected five plant extracts to test their efficacy in diabetic pregnancy in rats.

### Concentration of vitamin C in different plant extracts

**Figure 4:**
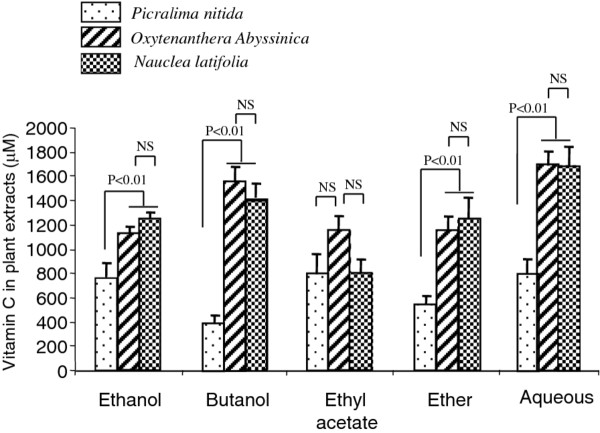
**Vitamin C concentration in plant extracts.** Vitamin C concentration was determined in a solution of 100 mg/ml of each extract as described in the Materials and Methods section. Results are mean ± SEM. Each value represents the mean of three determinations.

It is interesting to remark that all plant extracts showed substantial content of vitamin C (Figure 4). However, *Picralima* exhibited the lowest level of vitamin C. Vitamin C concentrations were quite similar in *Nauclea* and *Oxytenanthera*.

### Phytochemical composition of the extracts

Phythochemical analysis shows that *Oxytenanthera abyssinica* and *Picralima nitida* are good sources of polyphenols (tannins, flavonoids, anthocyans and quinonic derivates) and steroids (*Oxytenanthera)*. *Nauclea latifolia*, *Oxytenanthera abyssinica* and *Picralima nitida* are also rich in alkaloids (Table 1).

### Plant effects on human T cell proliferation

We used trypan blue exclusion test to assess the toxicity of plant extracts on T cell viability. We observed that plant extracts exhibited cytotoxic effect beyond the concentration of 50 μg/ml. Since no cytotoxic effect was observed from 5 μg/ml to 20 μg/ml, we then used three concentrations of plant extracts: 5 μg/ml, 10 μg/ml and 20 μg/ml.

**Figure 5:**
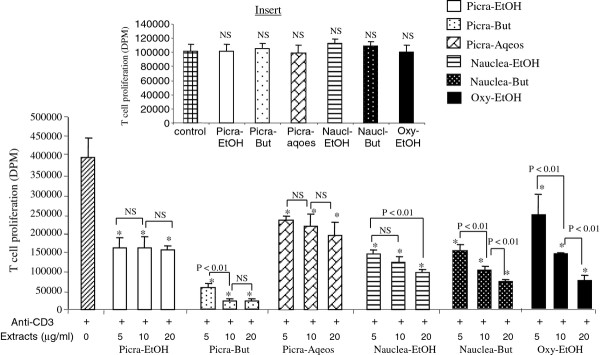
**Effects of plant extracts on T cell proliferation.** Figure 5 shows the effects of plant extracts on human T cell proliferation. Each value represents the mean of six determinations. Values are the means ± SEM. (*) indicates significant difference compared with the only anti-CD3-stimulated cells (p < 0.01). NS = non-significant difference. Figure 5 insert shows the effects of different extracts at 20 μg/ml without stimulation by anti-CD3. Picra-EtOH: ethanolic fraction of *Picralima nitida*; Picra-But: butaniolic fraction of *Picralima nitida*; Nauclea-EtOH: ethanolic fraction of *Nauclea latifolia*; Nauclea-But: butanolic fraction of *Nauclea latifolia*; Oxy-EtOH: butanolic fraction of *Oxytenanthera abyssinica*; Picra-Aqos: aqueous fraction of *Picralima nitida*.

The plant extracts significantly inhibited T cell proliferation activated by anti-CD3 antibody (Figure 5), in a dose-dependent manner, except in cases of Picra-but and Picra-aqueous which similarly inhibited T cell proliferation at the three concentrations. The highest inhibitory effect was observed at the concentration of 20 μg/ml with Picra-EtoH. The insert of Figure 5 shows that in the absence of anti-CD3 stimulation, plant extracts at concentration of 20 μg/ml did not significantly influence T cell proliferation.

### Fatty acids composition in different plant extracts

General analysis of Table 2 shows that all of the plants appeared to be good source of saturated (14:0, 16:0 and 18:0) and monounsaturated (16:1 and 18:1) fatty acids. However, *Nauclea latifolia and Oxytenanthera abyssinica* were richer in saturated fatty acids than *Picralima nitida* which was richer in monounsaturated (16:1 and 18:1) fatty acids than the former plants. Interestingly, none of the plant extracts exhibited omega-6 polyunsaturated fatty acids (like linoleic acid 18:2n-6 and arachidonic acid 20:4n-6) except ether extracts of all plants which curiously exhibited substantial levels of linoleic acid (LA), the precursor of arachidonic acid (AA). Moreover, all plant extracts showed moderate level of linolenic acid (C18:3n-3), except the residual aqueous fractions and ether fraction of *Nauclea latifolia*. The three principal fatty acids (*ie* AA, EPA and DHA) could not be detectable in none of the plant extracts.

## Discussion

More and more interest is now growing about plant use as an alternative therapy for lowering blood glucose levels in patients with diabetes mellitus. In the present study, we aimed to examine the efficacy of three different antidiabetic plants on hyperglycemia during diabetic pregnancy in rats. In this study, we induced a mild type 1 diabetic pregnancy in *wistar* rats by streptozotocin (STZ) injection. STZ when administered at a high single dose induces diabetes by the direct toxic effects on pancreatic β-islet cells [23]. However, when STZ is administered at low doses during 5 consecutive days, it induces mild type 1 diabetes, through a T-lymphocyte-dependent process, an autoimmune destruction of pancreatic β cells mediated by both CD4+ and CD8+ T cells [24,34]. Type 1 diabetic pregnancy induced in pregnant rats starting on day 5 of gestation, mimicking type 1 diabetes developed in human beings [24,35,36], is also well established [19-22] and represents a good model for several reasons [24,35,36]. Indeed, maternal STZ administration before pregnancy affects fertility and impairs embryo development during the pre-implantation period [37]. However, induction of diabetes by STZ injection on day 5 of gestation has no effect on embryo development [38].

In this study we observed that four different extracts exerted significant blood glucose lowering effect in pregnant diabetic animals. Ethanolic and butanolic extracts of *Picralima nitida*, butanolic extract of *Nauclea latifolia* and ethanolic extract of *Oxytenanthera abyssinica* significantly decreased glycemia in diabetic animals, as compared to untreated diabetic rats. Our results on *Picralima nitida* are in accordance with those reported by Aguwa *et al.*[15] and Inya-Agha *et al*. [16] who also demonstrated anti-hyperglycemic effects of *Picralima nitida* in alloxan-induced diabetic rats and rabbits. In contrast, Igboasoiyi *et al*. [14] have reported that seed extract of *Picralima nitida* did not possess hypoglycemic activity in alloxan-induced diabetic rats. This discrepancy could be related to the nature of solvent used and the mode of preparation of the plant extracts. Indeed, anti-hyperglycemic effects have been observed by Inya-Agha *et al.*[16] by treating diabetic animals with methanolic extracts of pulp, rind and seeds of *Picralima nitida*. Aguwa *et al.*[15] have also observed positive effect by using aqueous seed extract of the plant in diabetic rabbits. However, Igboasoiyi *et al*. [14], who did not observe any hypoglycemic activity in diabetic animals, have used total brut water extract of seeds. It is noteworthy that plant extracts did not either decrease or increase glycemia of the control animals, suggesting that plant extracts do not modulate glycemia under normal condition. These observations are in analogy to the results of some investigators who have observed that *Picralima nitida* extract [14] or *Mangifera indica*[39] failed to influence glycemia in normal control rats.

Interestingly, our results revealed that treatment of diabetic animals by these four extracts significantly diminished vitamin C concentration in the animals to the level of those treated with insulin. Moreover, plasma vitamin C concentrations were positively correlated with glycemia in diabetic animals (*R*^*2*^ = 0.98). These observations may be explained by the fact that L-ascorbate (vitamin C) is known to be synthesized from glucose by D-glucuronic acid pathway in mammals [40].

Beneficial effects of these plants may be related to the presence of biologically active compounds [5,41]. We have recently demonstrated that, *Zizyphus lotus* L. (Desf.), a plant known for its antidiabetic properties is a good source of antioxidant agents and its decoction exerts immunosuppressive activities [9]. We observed that extracts of *Nauclea latifolia, Oxytenanthera abyssinica* and *Picralima nitida* exhibited substantial antioxidant capacity. *Picralima* butanolic extract exerted the highest antioxidant activity. It is possible that the antioxidant activity might be due to the presence of vitamins. However, in the present study, there were not good correlation between vitamin C levels and antioxidant activities of plant extracts. Vitamin C levels were poorly correlated with antioxidant activity in ethanolic (*R*^*2*^ = 0.28), butanolic (*R*^*2*^ = 0.34) and ether (*R*^*2*^ = 0.04) extracts of plants. In contrast, vitamin C was negatively correlated with antioxidant activities in acetate (*R*^*2*^ = 0.97) and aqueous (*R*^*2*^ = 0.99) extracts of plants. Our results did not support those of Lenucci *et al*. [42] who have demonstrated that antioxidant activity is likely to be due to the presence of ascorbic acid, tocopherol and pigments. It is also possible that the antioxidant activity of extracts might be due to the presence of polyphenols as suggested by Lamien-Meda *et al.*[43]. Indeed, our results revealed that extracts of *Oxytenanthera abyssinica* and *Picralima nitida,* which exhibited the highest antioxidant capacity contained high level of polyphenols such as tannins, flavonoids, anthocyans, leucoanthocyans and quinonic derivates. These observations are in agreement with the findings of some investigators who have recently concluded that the high level of polyphenols in the fruit of *Zizyphus mauritania* (L.) was responsible for its antioxidant property [43].

Since antioxidants have been reported to modulate immune system implicated in type 1 diabetes [27], we examined the effects of the plant extracts on T cell proliferation. We observed that all the analyzed extracts (from the three plants) exerted an immunosuppressive effect on T cell proliferation. Our results support our previous findings [9] and those of Domingues *et al.*[44] who have reported that aqueous-ethanol extract of *Uncaria tomentosa*, a plant which belongs to the same family with *Nauclea latifolia* (Rubiaceae) significantly inhibited T lymphocyte proliferation and promoted immunological polarization toward a Th2 cytokine profile. Other investigators have also demonstrated the antiiflammatory activity of *Picralima nitida*[11]. To our knowledge, the present study is the first which investigated and demonstrated the immunosuppressive action of *Oxytenanthera abyssinica* on T cell proliferation. In this study butanolic extract of *Picralima nitida* appeared as the most potent immunosuppressor. At 10 and 20 μg/ml, the extract inhibited 94 ± 2.2% of T cell proliferation while at the same concentrations the other extracts inhibited T cell proliferation by 60.9 ± 7.6% (Picra-EtOH), 51.5 ± 2.2% (Picra-aqeos), 75.3 ± 3.5% (Nauclea-EtOH), 82.2 ± 2.9% (Nauclea-But) and 81.7 ± 8.5% (Oxy-EtOH). Generally, our results showed that the most potent immunosuppressor extract, Picra-But exhibited the most antioxidant activity, the most anti-hyperglycemic effect in diabetic rats but the less vitamin C content. These observations suggest that the significant immunosuppressive effect of Picra-But cannot be attributed to its content of vitamin C. Hence, it is possible that fatty acids might be responsible of the observed immunosuppressive effect. Indeed, among the three plants tested on T cell proliferation *P. nitida* and *O. abyssinica* are two richest plants that contains high level of fatty acids and they contained notably the well-known immunosuppressive omega-3 fatty acid, alpha-linolenic acid (LNA, C18:3n-3), an essential fatty acid and the precursor of eicosapentaenoic acid (EPA, C20:5n-3) and docosahexaenoic acid (DHA, C22:6n-3). It has been well established that n-3 fatty acids exert immunosuppressive and anti-inflammatory activities both in experimental and clinical studies [45]. In fact, fatty acids have been found to interfere with cell signalling particularly with the cascade of MAP kinases like ERK1/2 and p38 [46]. In the same way, da Conceição *et al.*[47] have shown that *Genipa americana* (a plant of Rubiacea family) fruit extract inhibited the proliferation of human trophoblast-derived BeWo cells by suppressing ERK and p38 MAPKs phosphorylation. However in the present study, it is noteworthy that the plant extracts, without mitogen stimulation, failed to inhibit cell proliferation suggesting that plant extracts do not influence T-cell proliferation under normal conditions. These results are in analogy to our previous observations that feeding omega-3 fatty acid enriched-diet to control animals did not significantly influence T-cell proliferation [19,20].

On the other hand, the immunosuppressive activity might also be attributed to the alkaloids contained in the plants. Our study showed that all of the plants (*Nauclea*, *Picralima* and *Oxytenanthera*), whose extracts exhibited a significant immunosuppressive action contained good level of alkaloids. Indeed, Duwiejua *et al*. [11] have demonstrated that pseudo-akuammigine, an alkaloid isolated from *Picralima nitida* seed extract possess anti-inflammatory and analgesic activity. Moreover, Bacher *et al.*[48] have demonstrated that oxindole alkaloids obtained from *Uncaria tomentosa* (a South American Rubiaceae) inhibited proliferation of acute lymphoblastic leukaemia cells.

The mechanism of action is likely to be that alkaloids in plants could interfere with T cell cycle. Indeed, an alkaloid-rich extract derived from *Nauclea latifolia* could interact *in vitro* with DNA of bacteria and mammalian cells leading to G2-M cell cycle arrest and heritable DNA-damage, as well as inducing *in vivo* single-strand breaks in liver, kidney and blood cells [49]. The findings by Mesaik *et al.*[50] have revealed that steroidal alkaloids from *Buxus hyrcana* exhibited suppressive effect on the phytohemagglutinin-stimulated T-cell proliferation, and that docking results revealed that these two alkaloids adopt a similar binding pattern at the active site of the IL-2. Yuan *et al.*[51] have demonstrated that rhynchophylline and isorhynchophylline, a pair of isomeric alkaloids of *Uncaria rhynchophylla* (a plant of Rubiacea family) suppressed phosphorylation of ERK and p38 MAPKs in microglial cells. Thus, by interfering with T cell cycle, alkaloids from antidiabetic plants could inhibit auto-reactive T cells, which lead to autoimmune type 1 diabetes.

## Conclusion

To our knowledge, the present study is the first carried out on the efficacy of antidiadetic plants in diabetic pregnancy. Different extracts of *Nauclea latifolia*, *Picralima nitida* and *Oxytenanthera abyssinica* bear potential therapeutic properties in diabetes and diabetic pregnancy as they possess antioxidant, anti-hyperglycemic and immunosuppressive activities. However, further studies are required to elucidate the effects of different extracts of plants in the modulation of different parameters related to diabetic pregnancy and autoimmune diseases.

## Abbreviations

Picra-EtOH: Ethanolic fraction of *Picralima nitida*; Picra-But: Butaniolic fraction of *Picralima nitida*; Nauclea-EtOH: Ethanolic fraction of *Nauclea latifolia*; Nauclea-But: Butanolic fraction of *Nauclea latifolia*; Oxy-EtOH: Butanolic fraction of *Oxytenanthera abyssinica*; Picra-Aqos: Aqueous fraction of *Picralima nitida*; MUFA: Monounsaturated fatty acids; SFA: Saturated fatty acids; PUFA: Polyunsaturated fatty acids; STZ: Streptozotocin.

## Competing interest

All of the authors have nothing to declare as far as the conflict of interest is concerned.

## Authors’ contributions

AY designed the study, wrote the manuscript and was in charge of major parts of the practical work. OG and JG participated in the technical work and contributed to the collection of plant materials and their extractions. AMS participated in the technical work. MM supervised the plant collection and extractions. AH contributed to the development of the protocol and participated in the interpretation of data. KM, ZT and NAK supervised the study and participated to the final drafts of the manuscript. KM, ZT and NAK established the collaborative and tripartite project CORUS2 between Dijon (France), Sousse (Tunisia) and Cotonou (Benin). All authors read and approved the final manuscript.

## Authors’ information

AY: PhD, Assistant Professor, Lecturer of Immunology and Cell Biology at Faculty of Sciences and Techniques, University of Abomey-Calavi, Benin.

OG: PhD, University of Burgundy, UPRES EA4183 Lipids and Cell Signaling, Faculty of Life Sciences, Dijon, France and Department of Physiology and Functional Exploration, University Hospital Farhat Hached, Sousse, Tunisia.

JG: Associate Professor of Chemistry at Faculty of Sciences and Techniques, University of Abomey-Calavi, Benin.

AH: Associate Professor of Physiology at INSERM U866, Université de Bourgogne.

AMS: Laboratory technician at INSERM UMR866, AgroSup/UB, Faculty of Life Sciences, University of Bourgogne, France.

ZT: Professor of Physiology at Department of Physiology and Functional Exploration, University Hospital Farhat Hached, Sousse, Tunisia.

MM: Professor of Chemistry at Faculty of Sciences and Techniques, University of Abomey-Calavi, Benin.

KM: PhD, Professor of Cell Biology at Faculty of Sciences and Techniques, University of Abomey-Calavi.

NAK: Professor of Physiolgy at Faculty of Life Sciences, University of Bourgogne France.

## Pre-publication history

The pre-publication history for this paper can be accessed here:

http://www.biomedcentral.com/1472-6882/13/77/prepub
